# Revealing the Function and the Structural Model of Ts4: Insights into the “Non-Toxic” Toxin from *Tityus serrulatus* Venom

**DOI:** 10.3390/toxins7072534

**Published:** 2015-07-06

**Authors:** Manuela B. Pucca, Felipe A. Cerni, Steve Peigneur, Karla C. F. Bordon, Jan Tytgat, Eliane C. Arantes

**Affiliations:** 1 Department of Physics and Chemistry, School of Pharmaceutical Sciences of Ribeirão Preto, University of São Paulo, Av. do Café, s/n, Ribeirão Preto, SP 14040-903, Brazil; E-Mails: manupucca@usp.br (M.B.P.); cerni@fcfrp.usp.br (F.A.C.); karla@fcfrp.usp.br (K.C.F.B.); 2 Toxicology and Pharmacology, University of Leuven, O&N 2, Herestraat 49, P.O. Box 922, Leuven 3000, Belgium; E-Mails: steve.peigneur@pharm.kuleuven.be (S.P.); jan.tytgat@pharm.kuleuven.be (J.T.)

**Keywords:** *Tityus serrulatus*, Nav1.6, envenomation, scorpion toxin, sodium channel

## Abstract

The toxin, previously described as a “non-toxic” toxin, was isolated from the scorpion venom of *Tityus serrulatus* (Ts), responsible for the most severe and the highest number of accidents in Brazil. In this study, the subtype specificity and selectivity of Ts4 was investigated using six mammalian Nav channels (Nav1.2→Nav1.6 and Nav1.8) and two insect Nav channels (DmNav1 and BgNav). The electrophysiological assays showed that Ts4 specifically inhibited the fast inactivation of Nav1.6 channels, the most abundant sodium channel expressed in the adult central nervous system, and can no longer be classified as a “non-toxic peptide”. Based on the results, we could classify the Ts4 as a classical α-toxin. The Ts4 3D-structural model was built based on the solved X-ray Ts1 3D-structure, the major toxin from Ts venom with which it shares high sequence identity (65.57%). The Ts4 model revealed a flattened triangular shape constituted by three-stranded antiparallel β-sheet and one α-helix stabilized by four disulfide bonds. The absence of a Lys in the first amino acid residue of the *N*-terminal of Ts4 is probably the main responsible for its low toxicity. Other key amino acid residues important to the toxicity of α- and β-toxins are discussed here.

## 1. Introduction

Scorpion envenoming is recognized as an important problem in specific tropical/subtropical areas of the world [1]. In Brazil, although 22 species of the *Tityus* genus have been described in the country, *Tityus serrulatus* (Ts) is responsible for the highest number of accidents and also the most severe [2].

Ts venom is comprised of several compounds such as mucus, salts, proteins with high molecular mass, nucleotides, lipids, amino acids, hyaluronidase, hypotensins, metalloprotease and neurotoxins [3,4,5,6,7,8]. Neurotoxins are the most thoroughly studied Ts venom components due to their clinical relevance and pharmacological action on ion channels [6,9,10]. Ts venom presents neurotoxins specific to sodium and potassium channels. Sodium channel specific toxins (NaTxs) are peptides of 61–76 amino acid residues with four disulfide bridges and based on their binding and physiological effect they can be classified into α- and β-toxins [11,12,13,14]. Moreover, these toxins are classified according to their binding site. The α-toxins can be divided into 3 groups: α-classic, α-insect and α-like. (a) The α-classic are highly active in mammals and their binding affinity to sodium channels is [15] reduced by membrane depolarization, (b) The α-insect show high toxicity towards Nav channels of insects and their binding to neuronal membranes is independent of membrane potential, and (c) α-like, that are active on both mammals and insects Nav channels, with a preference for insects [16]. On the other hand, β-toxins are classified according to their pharmacological preference for insect and mammalian Nav channels into four groups. (a) βm, active on mammalian Nav channels; (b) βi, that selectively act on insect Nav channels; (c) β-like, for those without preference between mammalian and insect Nav; and (d) βα, for toxins that present primary structure of β-toxins, but with a functional α-effect [17]. Potassium channel specific toxins (KTxs) are peptides of 23-64 amino acid residues with three or four disulfide bridges and are defined by the presence of the cysteine-stabilized α/β motif, in which the disulfide bridges covalently link a segment of α-helix with one strand of the β-sheet structure [18,19].

Until now, several Ts neurotoxins identified have been classified as NaTxs or KTxs. Ts1 and Ts1-G are β-NaTxs [20,21]; Ts2, Ts3, Ts5, Ts17 and Ts18 are α-NaTxs [8,15,22,23]; Ts6, Ts7, Ts9, Ts15 and Ts16 are α-KTxs [15,24,25,26]; and Ts8, Ts19 and Ts19 Frag-I and II are β-KTx [8,15] (P86822). Nevertheless, several Ts toxins need further studies to be classified such as Ts4.

Ts4 (also named TsTX-VI, Tityustoxin-6, Tityustoxin VI, TsTXVI, Toxin VI, Ts VI and TsNTxP) is composed of 62 amino acid residues and four disulfide bridges [27]. It presents high identity (65.57%) with Ts1, the major and the most known toxic peptide from Ts venom. Nonetheless, it is considered non-toxic to mice because of being unable to induce the characteristics symptoms of toxicity produced by other scorpion toxins [28]. On the other hand, Ts4 can induce an allergic reaction with lachrymation, spasm of the hind legs of mice and the production of dose dependent neurotransmitter liberation (GABA and Glu) of synaptosomes [29], which suggested that Ts4 does have an effect on mammalian cells. Therefore, the aim of this study was to reveal the functional characteristics of Ts4, by means of electrophysiological characterization. Further, using structural modeling we identified some key amino acid residues that could explain the low toxicity of Ts4.

## 2. Results

### 2.1. Ts4 Was Successfully Purified

**Figure 1:**
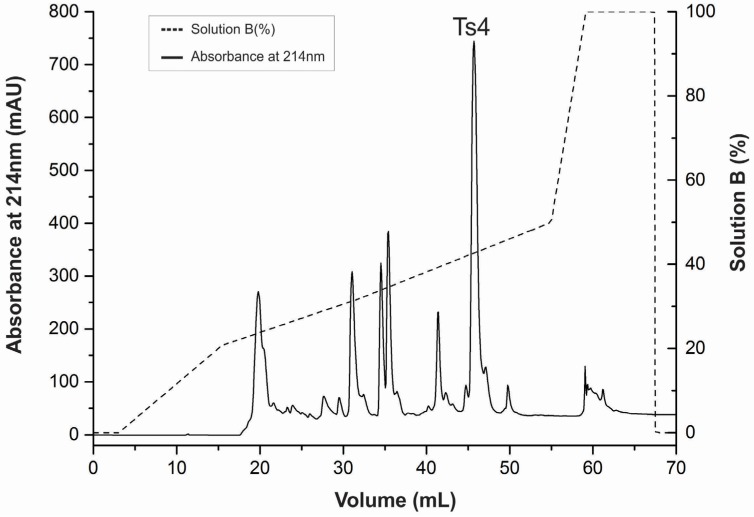
Reversed-phase FPLC of fraction VIIIB resulting from the Ts venom fractionation procedure. The fraction VIIIB was submitted to a reversed-phase chromatography on a C18 column (4.6 mm × 250 mm, 5 μm particles) equilibrated with 0.1% (*v*/*v*) of trifluoroacetic acid (TFA). Adsorbed proteins were eluted using a concentration gradient from 0% to 100% of solution B (80% acetonitrile in 0.1% TFA), represented by the dotted line. Flow: 0.8 mL/min. Absorbance was monitored at 214 nm, at 25 °C.

The *T. serrulatus* venom was fractionated using the improved CM-cellulose method described by Cerni *et al*., (2014). Venom fraction VIIIB was further submitted to RP-FPLC in an Äkta Purifier UPC-10 system. The major peak (>700 mAU) was determined as Ts4 (Figure 1) with a recovery percentage of 0.42% of the total venom. The mass spectrometry and sequence results confirmed the purity of the toxin and the mass (6703.948) of the mature toxin Ts4.

### 2.2. Ts4 Acts as an α-Toxin on Nav1.6 Channel

**Figure 2:**
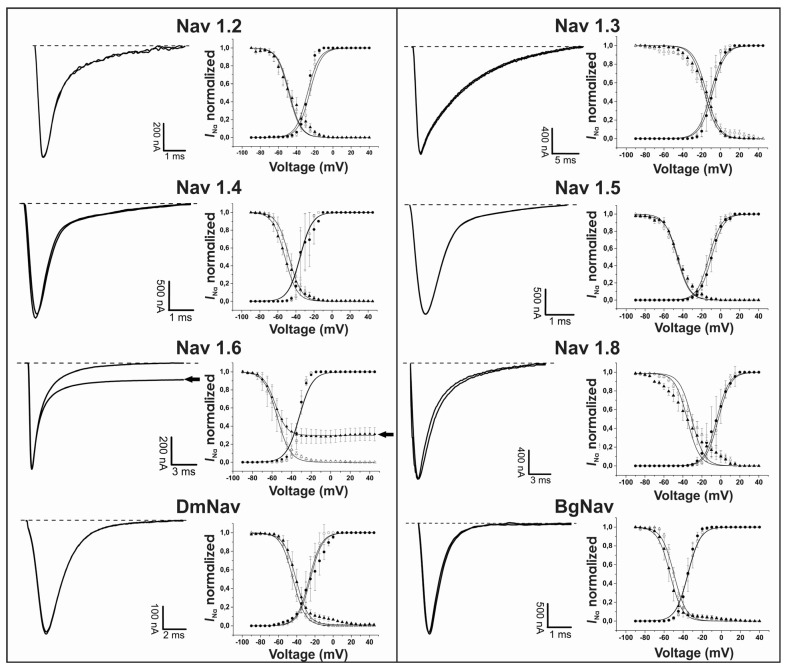
Electrophysiological screening of the effect of Ts4 on 8 different cloned voltage-gated sodium channels. Left panels: representative whole-cell current traces. Right panels: effects of Ts4 on the voltage dependence of steady-state activation and inactivation. Control conditions (open symbols) and after the addition of 500 nM Ts4 (closed symbols). Activation is shown by circles and inactivation by triangles.

**Figure 3:**
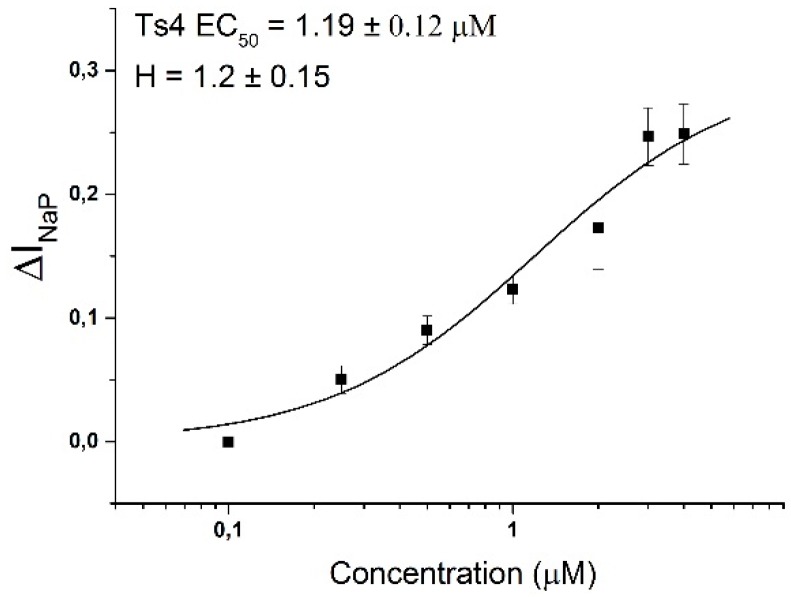
Ts4 concentration-response curve on Nav1.6. The concentration-response curve was performed using the ∆*I*_NaP_. The calculated EC_50_ value is shown above the curve. Data are presented as the mean ± SEM (*n* ≥ 3). The data were fitted with the Hill 1 equation.

The electrophysiology of Ts4 shows that the toxin specifically inhibited the fast inactivation of Nav1.6 with no effect on all the others Nav channels tested: Nav1.2, Nav1.3, Nav1.4, Nav1.5, Nav1.8, BgNav1 and DmNav1 (Figure 2, left panels). Application of 500 nM of Ts4 on Nav1.6 resulted in non-inactivating currents amounting 30% ± 3% of the *I*_Na_ peak amplitude with an alteration in the steady-state inactivation curve. No shift of activation and steady-state inactivation curves was observed for the other channels (Figure 2, right panels). To assess the concentration dependence of the Ts4-induced effects on Nav1.6, a dose-response curve was constructed, in which the percentage of current inhibition was plotted as a function of the concentration (Figure 3). Nav1.6 EC_50_ values yielded 1.19 ± 0.12 μM. The toxin induced effect on Nav1.6 was reversible upon wash-out (data not shown).

### 2.3. Ts4 3D-Structural Model and Amino Acids Implicated in Activity

The homology model of Ts4 was built based on the X-ray structure of Ts1 (Protein DataBank ID 1NPI). The Ts4 was found to be suitable for the homology modeling, because in general, 30% sequence identity is required for generating useful models [30] and here, the sequence alignment identity score was 65.57% with homologous template sequence.

The PDB file of Ts4 model was produced using three different web servers (SWISS-MODEL, I-TASSER and 3D-JIGSAW) to search for the best model.

Ts4 SWISS-MODEL PDB file automatically eliminated the *N*-terminal Gly1 to fit better the template model, Ts1 (Ramachandran Plot: 98.3% of the residues in favored region; 1.7% in allowed region). On the other hand, Ts4 I-TASSER PDB file showed low structure validation (Ramachandran Plot: 85%). Therefore, both models were discarded.

The best Ts4 Model was generated by the confident web server, 3D-JIGSAW, previously used [31,32,33,34]. Using 3D-JIGSAW we also constructed two different models for Ts4 based on the two deposited amino acid sequences (O77463* and P45669^#^), which present a unique amino acid substitution at 50th position (Asp* or Glu^#^). However, as expected, the model constructed with the sequence of Ts4 found in the venom (P45669^#^) and used in this study generated the best model (Ramachandran Plot: 78.3% residues in favored region and 98.3% residues in favored region/1.7% in outliner region—Trp40 for O77463* and P45669^#^, respectively).

Furthermore, the PDB 3D-JIGSAW Ts4 model (P45669^#^) was energy minimized with YASARA and finally validated with different web-based tools: Verify-3D, ERAAT, ProSA-web and Ramachandran Plot. The VERIFY 3D^a^ program validates the structure by a statistical approach that measures the compatibility of an amino acid sequence with the model structure [35]. ERRAT^b^ calculates the structure error function based on the statistics of non-bonded atom-atom interactions (compared to a database of reliable high-resolution structures) [36]. ProSa-web^c^ calculates an overall quality score for a specific input structure along with Z-score [37]. The Ramachandran Plot^d^ shows the phi-psi torsion angles for all residues in the structure (except those at the chain termini) [38]. The profile score for Ts4 model was 80%^a^ (0.2 in 3D/1D profile); ERRAT quality 100^b^; Z-score of −4.67^c^ and Ramachandran residues in favored regions 98.3%^d^; all which correspond to acceptable environment of the model (each superscript letter was added to indicate which software was used to obtain the profile score).

**Figure 4:**
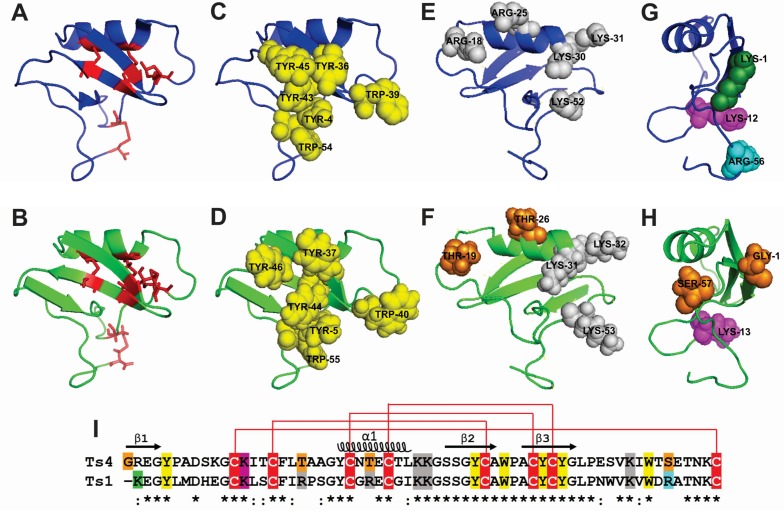
Cartoon representation of Ts1 structure and Ts4 3D-structural model. The cartoons represent the flattened triangular shape structure of the α-helix/β-sheet motif (CSαβ) of Ts1 (blue) and Ts4 (green). The Ts4 different amino acid residues compared to Ts1 are highlighted in orange. (**A**,**B**) Face A (front view): Cysteine residues are shown as red stick. (**C**,**D**) Face A (front view): Conserved aromatic cluster (yellow). (**E**,**F**) Face B (back view): Important structural positive residues (grey). (**G**,**H**) Face C (side view): Residues implicated in voltage-gated sodium channel (Nav) activity. *N*-terminal amino acid residue (green). Residue implicated in β-toxin activity (magenta). Residue implicated in α-toxin activity (cyan). (**I**) Sequence alignment of Ts4 and Ts1. The alignment of the Ts4 and Ts1 were created by Clustal Omega version 2.1. (*) identical residues; (:) highly conserved residues. Cysteine residues are highlighted in red. The residues are following (**A**–**H**) coloring patterns.

Ts4 model shows a three-stranded antiparallel β-sheet (residues 1–4, 34–39 and 42–47) and an α-helix (residues 23–30) (Figure 4). Ts4 presents an identical disulfide bridge pattern like Ts1, confirming that the model was satisfactory (Figure 4A,B). The α-helix is linked to the β-strand 3 by two disulfide bonds Cys24–Cys43 and Cys28–Cys45. The third disulfide bridge, Cys16–Cys38, links the loop 1 between the β-strand 1 and the α-helix. The fourth disulfide bridge, Cys12–Cys62, links the loop 1 and the *C*-terminus.

Ts4 showed a flattened triangular shape with two faces (A and B). Moreover, similar to Ts1, the central part of Ts4 face A presents the “conserved hydrophobic surface” or the “herring bone motif” represented by the aromatic residues: Tyr5, Tyr37, Trp40, Tyr44, Tyr46 and Trp55 (Figure 4 C,D,I).

However, we observed some differences among the important positive amino acid residues in the face B (Figure 4 E,F,I). Ts1 presents the residues Arg18, Arg25, Lys30, Lys31 and Lys52, which correspond in Ts4 to Thr19, Thr26, Lys31, Lys32 and Lys53.

Concerning the amino acid residues considered to play a key role for Nav activity, Ts1 presents a Lys1, Lys12 and Arg56, which correspond in Ts4 to Arg21, Lys13 and Ser57 (Figure 4G,H,I).

## 3. Discussion

*T. serrulatus* venom has been fractionated and studied in the last six decades [39,40,41]. Nevertheless, using the information of venom Ts glands [8], we know that the components already isolated and functionally studied are minor compared to the rich content of the crude venom.

Ts4 was one of the Ts toxins identified by pioneers in the Ts venom [27,29]. Intriguingly, Ts4 presents high identity with Ts1 (65.57%), the most known toxic component in the venom (LD_50_ = 76 ± 9 µg/Kg) [42]. However, based on the described non-toxic potential of Ts4, most of the studies found in the literature with this toxin aimed at the production of scorpion antivenoms [43,44,45,46]. Ts4 is capable of eliciting rabbit anti-peptide antibodies which recognize and neutralize *in vitro* the lethal effects of Ts venom and several Ts toxins [46,47]. This observation clearly indicates a structural resemblance between Ts1 and Ts4, as also evidenced by our structural model.

Knowing that non-toxic peptides stand out for their high interest in the pharmaceutical industry when having therapeutic effects [48], here we proposed to investigate electrophysiologically the Ts4 toxin.

The electrophysiological profile of Ts4 was assayed on cloned mammalian and insect Na_V_ channels expressed in *X. laevis* oocytes, revealing an α-effect on Nav1.6 channels. Ts4 specifically affected the inactivation of Nav1.6 channels with none effect on the voltage dependence of channel activation, pointing to a site 3 binding toxin. Based on this, we classified the Ts4 as a classical α-toxin. Indeed, the absence of any effect (*i.e*., no slowing of inactivation kinetics) on insect Nav channels, confirms that Ts4 is a classical α-toxin and not an α-like [16]. As expected, the electrophysiological data of Ts4 was completely differing from the ones described for other sodium channels toxic peptides from Ts venom. (*i*) Ts1 exerts a plethora of pharmacological effects on different Nav channel: it interferes with the activation process and also modulates the inactivation in a bell-shaped voltage-dependent matter. In respect to Nav1.6 isoform, Ts1 influenced the kinetics of activation with a leftward shift and also significant leftward shift the steady-state inactivation [20]. (*ii*) Ts2 inhibits rapid inactivation of Nav1.2, Nav1.3, Nav1.5, Nav1.6 and Nav1.7, and significantly shifts the voltage dependence of activation of Nav1.3 channels [22]. (*iii*) Ts5 inhibits the rapid inactivation of the mammalian sodium channels Nav1.2, Nav1.3, Nav1.4, Nav1.5, Nav1.6, Nav1.7 and DmNav1 [23]. This observation stresses that Ts4 is the only Ts toxin with a unique specificity. In comparison with the venom of another species from the same genus (*T. fasciolatus*), Ts4 shows high identity with the toxin Tf4 (77%), which is also classified as an α-toxin using the “single sucrose-gap” technique in frog nerves (*Rana catesbeiana*) [49]. Therefore, we assume that the specificity of Ts4 for a unique channel (Nav1.6) consequently results in its low toxicity leading to its classification of a “non-toxic” peptide.

The Nav1.6 channel is the most abundantly sodium channel expressed in the adult central nervous system [50]. Moreover, there is evidence for a role of Nav1.6 in facilitating increases in neuronal hyperexcitability during epiloptogenesis [51]. This is also supported by current treatments for epilepsy which aim at blocking seizures through medications which reduce sodium current [52]. Indeed, some sporadic cases of epileptic seizures may occur in up to 5% of individuals, especially children, after scorpion stings. Concerning *T. serrulatus* species, a case of long-lasting brain lesion with seizures occurring approximately once monthly has been reported [53].

Our electrophysiological results with Ts4 corroborate with the previous study using mice challenged with this toxin where contractions of hind legs were observed in the animals [29] and, undoubtedly, epilepsy can cause body jerks and spasms [54].

In this way, the question still remains why epilepsy is not observed often when *T. serrulatus* venom or Ts toxins (Ts2, Ts4 and Ts5—proven to be α-toxins on Nav1.6) are administered?

Several answers could be brought forward such as: (1) The potency of the venom (age of the scorpion, the quantity of venom injected and the localization of the sting); (2) the age of the victim and the body weight; (3) the hepatic and renal clearance of the venom proteins; (4) the low diffusivity of toxins in the absence of hyaluronidase and finally (5) the blood-brain barrier (BBB), probably the most convincing justification. BBB selectivity could be the main responsible preventing epilepsy during scorpion envenoming. Although some studies show a BBB molecular mass cut-off of 400 or less [55], others present that there are more parameters involved in the membrane permeability such as lipophilicity, pKa, hydrogen bonding and biological factors [56,57]. To date, only a small number of known venom components can penetrate the BBB, among which chlorotoxin and apamin being the most widely known [58,59]. However, it is estimated that there are many venom components of which capability to penetrate BBB have not yet been assayed. We cannot prove that Ts4 can reach the Central Nervous System (CNS) but we cannot discard this possibility either. However, our study certainly supports the idea that Ts4 could provoke CNS alterations and/or epileptic episodes if it was intracerebroventricularly administrated.

Additionally, based on the previous observed Ts4 allergic symptoms in mice (e.g., lachrymation), we also speculate that this toxin can activate mast cells. The literature shows that venoms and toxins can activate the cells from the immune system. Further, several Ts toxins have been immunologically investigated [60,61,62,63,64,65]. However, most of these studies demonstrate the involvement of Ts toxins in the inflammatory response focusing mainly on macrophages. Indeed, the presence of Nav1.6 has already been described intracellularly in macrophages. However, how these toxins could reach the receptor in the cytosol or endoplasmic reticulum (ER) is still obscure [23,66]. In respect to mast cells, the presence of Nav1.6 has not been reported; however, these cells present toll-like receptors (TLRs) which are known to be activated by Ts toxins [60]. In any case, independent of the mechanism of action (via sodium channel Nav1.6, TLR or other receptor), we believe that Ts4 affects the mast cell function resulting in an allergic reaction. Indeed, a study recently shows that mast cells are an important intermediate between neuropeptide release and the inflammatory cascade during pulmonary hyperresponsiveness induced by the venom of the scorpion *Androctonus australis hector* [67].With this in mind, we encourage further studies to investigate the activation of mast cells induced by Ts4.

In an attempt to understand the relationship between Ts4 structure and function, we also performed an alignment and a 3D model of Ts4 based on Ts1 sequence and crystal structure.

The model of Ts4 revealed an α-helix/β-sheet motif (CSαβ) [68]. Further, Ts4 presented a flattened triangular shape with two faces as previously defined [69]. Identical to Ts1, Ts4 presented the face A covered by aromatic amino acid residues, namely conserved hydrophobic residues [70]. Ts4 also presented amino acid residues structurally important (Tyr5, Gly36, Tyr37 and Leu48) which are also found in Ts1.

**Figure 5:**
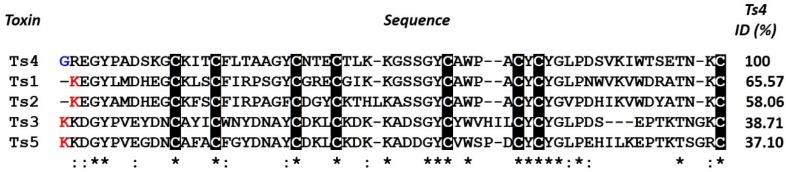
Multiple sequence alignment of sodium channel Ts toxins (Ts1-Ts5). The alignment of the Ts1, Ts2, Ts3, Ts4 and Ts5 and identity (%) were created by Clustal Omega version 2.1. (*) identical residues; (:) highly conserved residues. Cysteine residues are highlighted in black. The Lys1 is in red and the Gly1 is in blue.

In respect to the differences between Ts1 and Ts4 amino acid residues, the presence of a Gly1 (non-charged residue) in Ts4 instead of Lys1 (basic residue) is very remarkable. Indeed, this can be the most important residue responsible for loss of toxicity compared to Ts1 since all long-chain toxins appeared to be sensitive to the modification of the *N*-terminal basic residue [14,71,72,73]. Based on atomic resolution of the X-ray crystallography of Ts1, another study also showed that the Lys1 is involved both in crystalline contacts and in anchoring the *C*-terminus via interactions with the toxin residues Val51, Asn49 and Leu47. Therefore, a change in the charge of the *N*-terminal amino acid residues could reflect directly on the position of the *C*-terminus (for more details see [74]). Indeed, compared with other proven sodium channel toxins isolated from Ts venom, Ts1-Ts5 (Ts17 and Ts18 were also classified as Nav toxins, but they were only described on transcriptome analysis [8]), Ts4 is the only one that does not present a Lys in the first position of the *N*-terminal, a quite remarkably observation (Figure 5). In our model, when Gly1 was automatically excluded from *N*-terminal by the SWISS-MODEL tool, 98.3% of the residues are in favored region and 1.7% are in allowed region; however, when Gly1 is considered (3D-JIGSAW), the Trp40, which corresponds to 1.7% of the number of residues, presents in the outlier region. Therefore, Gly1 clearly causes a modification in the 3D-structure of Ts4 and probably is the main responsible for its low toxicity. It has also been shown that chemical modification of the *N*-terminal residues Lys1 in Ts1 [71], Arg1 in AaHI and AaHIII [14,72], Val1 and Arg2 in AaHIII [72] and the amino group in AaHI [72] produces weakly active or inactive derivatives. Cleavage of the six *N*-terminal residues of Ts1 or five *N*-terminal residues of BomIII gives non-toxic derivatives [74]. Other amino acid residue relevant to the Ts4 3D-structure is the Glu50 (instead of the Asp50 identified by transcriptome analysis). In the structure modelled using Asp50, 6.7% of the residues are in the outlier region (four amino acids residues). On the other hand, when the Glu50 (identified by venomic analysis) was used to model, only one amino acid residue (Trp40), which corresponds to 1.7%, was present in the outlier region.

Concerning toxicity of scorpion toxins, other key amino acid residues can be important to. Lys12 seems to be very important in β-toxins [73] and Lys/Arg56 in α-toxins (Ts1 numbering) [14,72]. Indeed Ts1 presents Lys12 and it is classified as a β-toxin; but it also has the Arg56 and does not present an α-effect on any channel [75]. Moreover, although Ts4 presents the Lys13 (corresponding to Lys12 in Ts1 numbering), we did not observe a β-effect. Additionally, Ts4 presents a non-charged residue (Ser57) instead of a basic residue (Arg56), with the latter seeing very important for the α-effect. Nevertheless, we still observed an α-effect induced by Ts4. Indeed, it is clear and remarkable the different position of these amino acids in the respectively structure (Face C: side view). Furthermore, Ts3 and Ts5 present Thr56 and Thr58, respectively, and they still show an α-effect on sodium channels, which could indicate that polar and uncharged side chain amino acids such as Ser and Thr can be important to α-toxin effect. Based on this observation, we argue that further studies are still necessary to understand the involvement of the important amino acids implicated to activity, especially regarding *T. serrulatus* toxins. Therefore, further structure-function studies combining Ts1 mutagenesis of the following amino acids: Lys1, Lys12, Arg18, Arg25 and Arg56 will be very interesting.

## 4. Experimental Section

### 4.1. Ts4 Isolation

*T. serrulatus* venom was obtained from the vivarium of School of Medicine of Ribeirão Preto, University of São Paulo, Brazil, using electrical stimulation method—12 mV [76].

Desiccated Ts venom (50 mg) was fractionated on a CM-cellulose-52 column connected to a Fast Protein Liquid Chromatography (FPLC) system, using the improved method described by Cerni and co-authors [24].

The fraction VIIIB eluted from CM-cellulose-52 Whatman^®^ (GE Healthcare, Uppsala, Sweden) was used to obtain Ts4. RP-FPLC of the fraction VIIIB was performed in an Äkta Purifier UPC-10 system (GE Healthcare, Uppsala, Sweden), using a 4.6 mm × 250.0 mm C18 column (Shimadzu Corp., Kyoto, Kansai, Japan) equilibrated with 0.1% (*v*/*v*) trifluoroacetic acid (TFA, Avantor Performance Materials Inc., Center Valley, PA, USA) at a flow rate of 0.8 mL/min. The samples were eluted with steps of concentration gradient from 0 to 100% of solution B (80% acetonitrile (Avantor Performance Materials Inc., Center Valley, PA, USA) in 0.1% TFA), at a flow rate of 0.8 mL/min. Absorbance was monitored at 214 nm. Pure toxins were lyophilized and stored at −20 °C.

The determination of *N*-terminal amino acid residues of Ts4 was performed by Edman degradation [77], on a Protein Sequencer model PPSQ-33A (Shimadzu Co., Kyoto, Kansai, Japan). To confirm the purity of the toxin, the mass of Ts4 was measured by MALDI-TOF/TOF UltrafleXtreme (Bruker Daltonics, Billerica, MA, USA) mass spectrometer. The spectrum was processed using using Flex Analysis software version 3.3.65 (Bruker Daltonics, Billerica, MA, USA, 2012).

### 4.2. Electrophysiological Experiments

#### 4.2.1. Sodium Channel Expression

For the expression of Nav channels of mammalian (Nav1.2, Nav1.3, Nav1.4, Nav1.5, Nav1.6, and Nav1.8) and insect (DmNav1 from the fruit fly *Drosophila melanogaster* and BgNav from the cockroach *Blattella germanica*) origin, the cRNA were synthesized from linearized plasmids using large-scale T7 or SP6 mMESSAGE-mMACHINE transcription kits (Ambion, Carlsbad, CA, USA).

The resected oocytes lobes from female *Xenopus laevis* frogs were used to obtain the stages V-VI oocytes. The frog surgery was performed in the Aquatic Facility at the KULeuven University and the use of the frogs is in accordance with the license number LA1210239. Immersion in Tricaine (Sigma Chemical Co, St. Louis, MO, USA) solution (1 g/L) was the anesthetic method used to induce anesthesia. The frog procedure was previously used [24,78,79].

Oocytes (stages V-VI) were injected with 30–60 nL of the different channels respectively, using a micro-injector (Drummond Scientific, Broomall, PA, USA). ND-96 solution was used for the oocytes incubation (in mM): 96 NaCl, 2 KCl, 2 MgCl_2_, 1.8 CaCl_2_, 5 HEPES (pH 7.4) supplemented with 50 mg/L gentamicin sulfate (Sigma Chemical Co, St. Louis, MO, USA) and 180 mg/L theophylline (Sigma Chemical Co, St. Louis, MO, USA).

#### 4.2.2. Electrophysiological Measurements

Sodium currents were recorded using the two-microelectrode voltage-clamp technique at room temperature (18–22 °C). The recordings were processed by GeneClamp 500 amplifier (Molecular Devices, Downingtown, PA, USA) controlled by a pClamp data acquisition system (Axon Instruments, Union City, CA, USA). Whole-cell currents from oocytes were recorded 1–7 days after injection. Currents and voltage electrodes had resistances from 0.7 to 1.5 MΩ and were filled with 3 M KCl. Currents were sampled at 20 kHz and filtered at 1 Hz using a four-pole low-pass Bessel filter. Leak subtraction was performed using a—P/4 protocol.

For the assays, 500 nM of Ts4 was added directly to the recording chamber from a stock solution of ND-96 to obtain the desired final concentration.

For the activation protocols, 100 ms test depolarization, ranging from −90 mV to +70 mV, were applied from a holding potential of −90 mV, in 5 mV increments at 5 s intervals. For the inactivation protocols, we employed double pulses, with a conditioning pulse applied from a holding potential of −100 mV to a range of potentials from −90 mV to 0 mV, in 5 mV increments for 50 ms, immediately followed by a test pulse to 0 mV (or −5 mV). Data were normalized to the maximal Nav current amplitude (*I_max_*), plotted against the pre-pulse potential and fitted using the Boltzmann equation: *I_Na_/I_max_ =* 1/[1+exp((*V_c_*−*V_h_*)/*k_h_*], where *V_h_* was the voltage corresponding to half-maximal inactivation, *V_c_* was the conditioning pre-pulse voltage, and *k_h_* was the slope factor. To assess the concentration-response relationships, data were fitted with the Hill equation: *y* = 100/[1 + (*EC_50_*/[toxin])*^h^*], where *y* is the amplitude of the toxin-induced effect, *EC_50_* is the toxin concentration at half maximal efficacy, [toxin] is the toxin concentration and *h* is the Hill coefficient.

Each experiment was performed at least 3-times. Data were analyzed using Clampfit 10.4 (Molecular Devices, Sunnyvale, CA, USA), Excel 2010 (Microsoft Corp., Redmond, WA, USA, 2010) and OriginPro 9.0 (OriginLab Corp., Northampton, MA, USA, 2012).

### 4.3. Amino Acid Sequence Alignment and Structural Modeling

Ts4 was aligned with the known Ts1 sequence using the National Center for Biotechnology Information (NCBI) Basic Local Alignment Search Tool (BLAST) software (http://clustalw.ddbj.nig.ac.jp/). The amino acid sequences were as follows: Ts1 (SCX1_TITSE; accession no. P15226), Ts4 (SCX4_TITSE; accession no. P45669 and O77463). To predict the Ts4 three-dimensional (3D) structure based on homology, we used bioinformatics tools, including the Protein Data Bank (PDB; http://www.rcsb.org/pdb). Homology modeling was performed using the target sequence and the solved X-ray 3D-structure of Ts1 [80]. The PDB template of Ts1 (1NPI) was uploaded to different web servers SWISS-MODEL (http://swissmodel.expasy.org/), I-TASSER (http://zhanglab.ccmb.med.umich.edu/I-TASSER/) and 3D-JIGSAW (http://bmm.cancerresearchuk.org/~3djigsaw/), to construct the Ts4 PDB file. The Ts4 PDB file was further energy-minimized with YASARA (http://www.yasara.org/minimizationserver.php). Ts4 PDB file utilized for the cartoon/spheres representation model was constructed using Pymol Molecular Graphics System (http://www.pymol.org/jymol). The validation for structure models was performed by using Verify-3D and ERAAT at Structural Analysis and Verification server (http://nihserver.mbi.ucla.edu/SAVES/); ProSA-web [38] at Protein Structural Analysis server (https://prosa.services.came.sbg.ac.at/prosa.php) and Ramachandran Plot analysis at University of Cambridge web server (http://mordred.bioc.cam.ac.uk/~rapper/rampage.php).

Model data are available in the Model Archive under modelarchive.org. The DOI (ma-a99q9) will be online after publication.

## 5. Conclusions

In conclusion, we reported for the first time the electrophysiological profile of Ts4 on 8 isoforms of Na_V_ channels. Ts4 was found to specifically inhibit the fast inactivation of Nav1.6 channels. Therefore, Ts4 can no longer be classified as a “non-toxic peptide”. The Ts4 alignment and model with Ts1 highlights some key amino acid residues that could be involved in the less toxic profile of Ts4. Hence, studying the scorpion toxins (e.g., Ts4) appears as a promising approach to understand the envenoming syndrome and to study target ion channels.
